# The Mirror of Erised: a retrospective population-wide study of Czech all-cause mortality data by COVID-19 vaccination status

**DOI:** 10.1186/s12889-025-23619-x

**Published:** 2025-07-10

**Authors:** Ondrej Vencalek, Tomas Furst, Elizabeth Princova, Jana Furstova

**Affiliations:** 1 Department of Mathematical Analysis and Application of Mathematics, Faculty of Science, Palacký University Olomouc, 17. listopadu 1192/12, Olomouc, 779 00 Czech Republic; 2 Olomouc University Social Health Institute, Sts Cyril and Methodius Faculty of Theology, Palacký University Olomouc, Univerzitní 244/22, Olomouc, 779 00 Czech Republic

**Keywords:** All-cause mortality, COVID-19, Vaccination status, Individual-level data, Healthy vaccinee effect, Healthy user bias, Vaccine effectiveness

## Abstract

**Background:**

In this study, we investigated the association between COVID-19 vaccination status and all-cause mortality (ACM) rate in the population of the Czech Republic between January 2020 and December 2022.

**Methods:**

In this retrospective study based on official population-wide individual (record-level) data, we analyzed monthly ACM rates stratified by COVID-19 vaccination status, sex, and age. The ACM was compared to expected mortality based on pre-COVID data. The recipients of the Janssen vaccine were excluded from the study. The final dataset comprised *N* = 5,636,949 individuals from the Czech Republic, encompassing all residents born between 1925 and 1980 who were alive on January 1, 2020.

**Results:**

Multiple peculiar patterns in ACM were revealed. The ACM of vaccinated individuals across several age cohorts was greatly diminished compared to the ACM of the unvaccinated, even in periods when virtually no COVID-19-related deaths were observed, suggesting a strong selection/indication bias. A similar drop in the ACM of newly vaccinated individuals was observed again during the booster campaign. With time from vaccination, the differences in ACM between groups with different vaccination statuses dwindled. Indication bias was observed at the beginning of the vaccination campaign when the frailest individuals were preferentially vaccinated.

**Conclusions:**

The population-wide data strongly suggest the presence of selection/indication bias, warranting careful interpretation of vaccination effectiveness estimates derived from observational studies.

**Supplementary Information:**

The online version contains supplementary material available at 10.1186/s12889-025-23619-x.

## Background

The COVID-19 pandemic has affected all levels of life. However, even five years into the pandemic, the availability of data for evaluating the effectiveness of individual measures applied to battle the pandemic is severely limited. For example, although a multitude of studies on vaccine effectiveness has been published (e.g. Cromer, et al. [1], Feikin, et al. [2], Soheili, et al. [3]), virtually none of them has made the source data openly available. Considering the push for open data and transparency in science [4, 5], this represents a serious flaw in the chain of evidence of vaccine effectiveness. Recently, however, the Institute of Health Information and Statistics of the Czech Republic (IHIS) [6] released population-wide, individual-level data on all-cause mortality (ACM) in relation to COVID-19 vaccination, which is, to the best of our knowledge, the only such dataset publicly available to date.

Since this dataset lacks information on the cause of death, it cannot be used to directly assess the protective effect of vaccination against COVID-19-related mortality. It can, however, be used for the comparison of ACM between vaccinated and unvaccinated populations. Previous studies revealed a peculiar pattern in partial datasets from individual Czech health insurance companies: The ACM of the vaccinated population was much lower (depending on age group) than that observed in the unvaccinated population, not only during COVID-19 waves, when the expected protective effect of the vaccines was expected to lead to such a result, but also in the summer of 2021 when virtually no COVID-19-related deaths were registered in the Czech Republic [7–9]. Based on these patterns, we hypothesized that a special case of healthy user bias, the so-called Healthy Vaccinee Effect (HVE, defined as the population opting for vaccination being healthier or more health-conscious [10]) was in play, causing a non-negligible bias in observational studies on COVID-19 vaccine effectiveness.

The previous studies [7–9], however, did not rely on population-wide data. The newly released dataset described here, therefore, presents an opportunity to perform a much more robust analysis. In this study, we aimed (i) to investigate the patterns in all-cause mortality across the population-wide data, and (ii) to discuss the possible causes and implications of these patterns.

## Methods

### Data

The principal dataset used in this paper was released by the Institute of Health Information and Statistics of the Czech Republic (IHIS) in 2024. The data contains over 11 million rows (*N* = 11,028,371) – a single row for each Czech resident who was alive on January 1, 2020, or was born during the period (from January 1, 2020, until December 31, 2022). For each individual, the row contains the year of birth, sex, exact date of death from any cause within the study period (for those who died) and exact dates, types and batch numbers of all COVID-19 vaccines given to that individual. To the best of our knowledge, this is the only officially released dataset that links all-cause mortality to COVID-19 vaccination status at the level of individuals on the scale of a whole country.

The following exclusion criteria were applied: (i) individuals with unspecified gender in the original dataset (*N* = 9,942); (ii) recipients of the Janssen vaccine (*N* = 410,057), as its primary course required only a single dose, whereas all other vaccines used in the Czech Republic required two doses; (iii) Czech residents born in 1980 or later, as these age groups accounted for only ~ 2% of all deaths during the study period and were therefore considered negligible for the purposes of this analysis; and (iv) Czech residents born before 1925, who were aged 95 or older at the beginning of this study. After applying these criteria, the final dataset included *N* = 5,636,949 individuals.

We have not found any significant discrepancies between this dataset and other publicly available summary statistics of the Czech population; thus, we have no reason to doubt the accuracy of the data. The dataset is available at https://github.com/PalackyUniversity/uzis-data-analysis.

Additional data on death counts and mortality rates in the period preceding the COVID-19 outbreak were acquired from the public database of the Czech Statistical Office. Specifically, mortality rates for 2019 were sourced from life tables constructed separately for males and females [11]. Seasonal effects were estimated using the data in Table 8 *− 7 Deaths by calendar month: 1950–2022*, included in the latest issue of *Czech Demographic Handbook – 2022* [12].

### Statistical analysis

Based on the above datasets, we provide a detailed account of the evolution of all-cause mortality rate in the Czech Republic between January 2020 and December 2022 with respect to sex, age, and vaccination status. No differentiation between COVID-19 vaccine types and batch numbers is performed (however, see Supplementary Table 1 for a detailed breakdown of vaccine types by dose). Since the data contains the exact date of vaccination, we consider individuals to be vaccinated with dose N from the day of administration of dose N until the day of administration of the dose *N* + 1 (or until his/her death or the end of the study period). Individuals vaccinated with dose three and higher are grouped into a single category.

First, ACM rates were computed for each sex, age category (determined by the decade of birth), month of the study period, and vaccination status by dividing the number of deceased in the category by the number of person-days spent by individuals in that category and converting this value to deaths per 1,000 person-years.

Next, the expected number of deaths in each cohort (separately for each individual year, i.e., 2020, 2021, and 2022) was computed based on the mortality rates in 2019. For each age (one-year bracket), the expected total number of deaths in the particular year was calculated by multiplying the number of individuals of that age at the beginning of the year by the 2019 mortality rate in the population of that age. Subsequently, the expected numbers of deaths were summed up for the entire 10-years cohort. The total expected annual number of deaths was then distributed across months based on the median percentage of deaths in each calendar month from 1950 to 2019, normalized to 100%. The resulting expected numbers of deaths and cohort sizes were subsequently used for the computation of the expected ACM rates.

Finally, prediction intervals were constructed, in which the empirical ACM values ​​should lie if the 2019 death rate were maintained (if no epidemic occurred). These intervals accounted for two sources of uncertainty, namely (i) the uncertainty inherent to the random binomial variable itself (i.e., the fact that the number of deaths that would occur in the respective year can differ from the expected value), and (ii) the interannual variability in the percentage of deaths in individual months (as calculated from the 70-years-long period of 1950–2019).

Considering these two sources of uncertainty, a 95% prediction interval was calculated using standard algorithm for its construction for the binomial distribution. To account for the interannual variability in the percentage of deaths for each calendar month, the 25th and 75th empirical quantiles from the 1950–2019 period were used as parameters for the binomial distribution. In other words, the 75th quantile was used as a parameter for this calculation for the upper bound of the prediction interval and the 25th quantile for the lower bound. The use of 25th and 75th quantiles instead of maximum and minimum values avoids bias caused, for example, by the occurrence of the past (e.g. influenza) epidemics. Note that the way in which annual total counts are distributed across months does not affect the 95% coverage of the total annual prediction interval.

## Results

Table 1 summarizes the descriptive characteristics of the study sample. There were slightly less men (*N* = 2,665,993; 47⋅3%) than women (*N* = 2,970,956; 52⋅7%). However, men accounted for a higher proportion of deaths during the study period (*N* = 190,934; 51⋅9%) compared to women (*N* = 177,251; 48⋅1%).

**Table 1:** Descriptive characteristics of the study sample; Czech republic, 2020−2022

			Deaths^b^	
Year of Birth	Sex	Size of cohort^a^	N	%
1925−1929	M	14,213	8,240	58⋅0
	F	38,006	20,832	54⋅8
1930−1939	M	130,345	46,108	35⋅4
	F	240,165	68,199	28⋅4
1940−1949	M	420,758	66,297	15⋅8
	F	563,450	52,370	9⋅3
1950−1959	M	603,083	44,848	7⋅4
	F	672,908	23,801	3⋅5
1960−1969	M	641,446	17,327	2⋅7
	F	635,315	8,191	1⋅3
1970−1979	M	856,148	8,114	0⋅9
	F	821,112	3,858	0⋅5

*M* Male, *F* Female

^a^At the beginning of the study period, on January 1, 2020

^b^During the study period, from January 1, 2020 to December 31, 2022

**Fig. 1:**
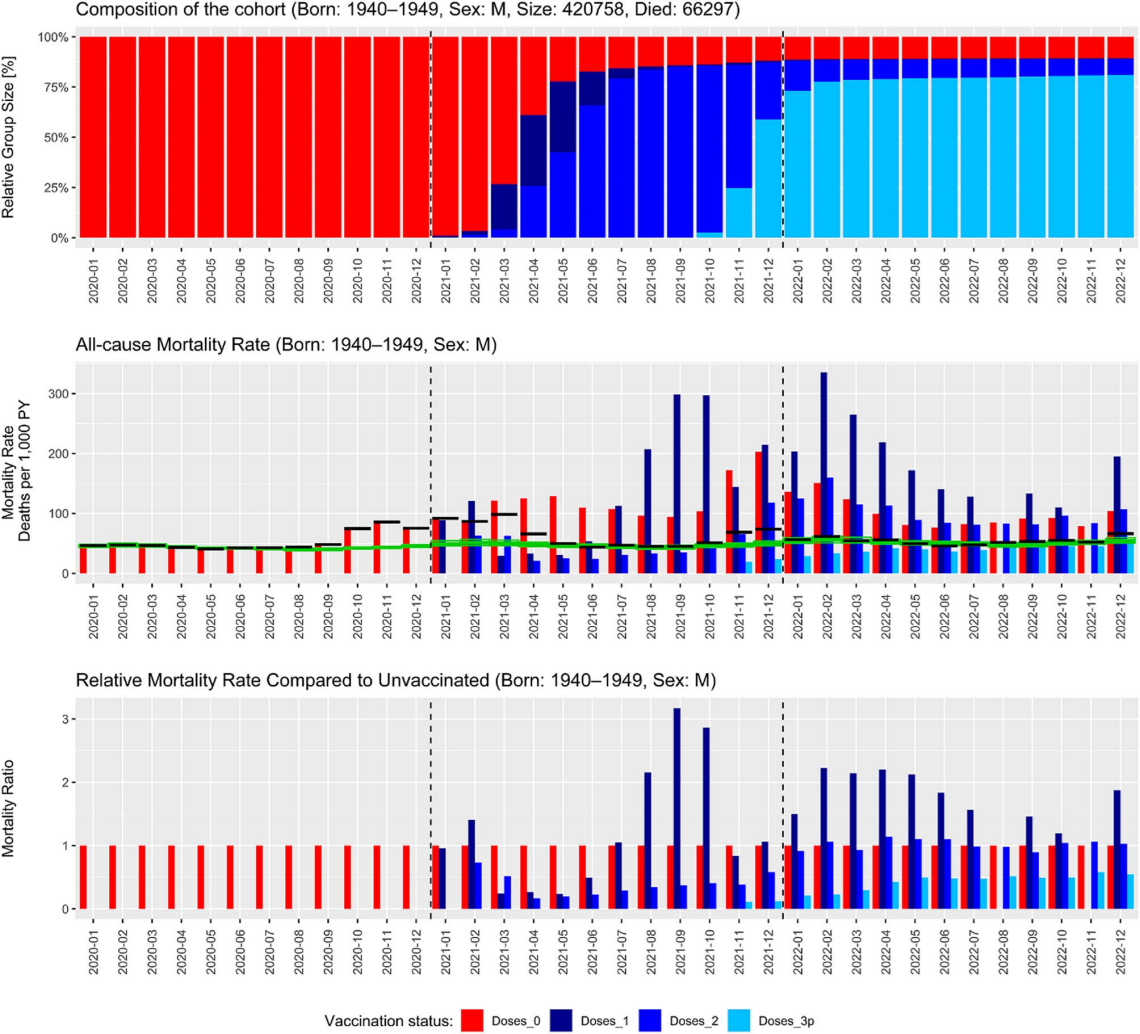
Evolution of the all-cause mortality (ACM) rate in the cohort of men born between 1940 and 1949; Czech Republic, 2020−2022 The top panel shows the relative composition of the population according to the vaccination status. The middle panel shows the ACM rate by vaccination status for each month between January 2020 and December 2022, the average ACM rate disregarding the vaccination status (black line) and the expected ACM rate (green line, see the Methods for details on the calculation). The bottom panel shows the ACM rates relative to the ACM rate of the unvaccinated. Vaccination status is color-coded as follows: Unvaccinated – red; individuals after a single dose of any COVID-19 vaccine – dark blue; individuals after two doses of any COVID-19 vaccine – blue; individuals after three or more doses – light blue. Note that mass vaccination for this group started on March 1, 2021; before that date, only the frailest individuals highlighted for preferential vaccination received the vaccine

**Fig. 2:**
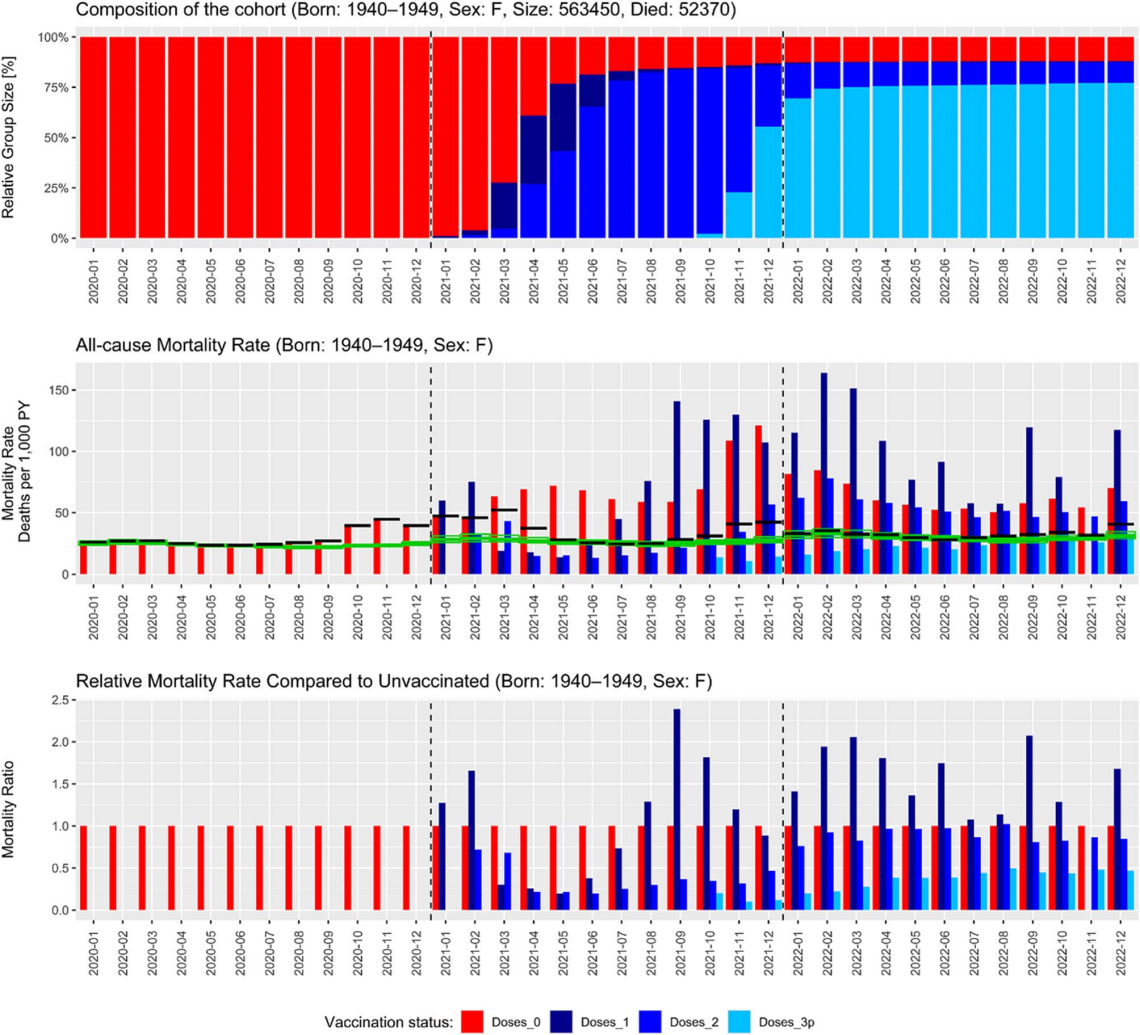
Evolution of the all-cause mortality (ACM) rate in the cohort of women born between 1940 and 1949; Czech Republic, 2020−2022 The top panel shows the relative composition of the population according to the vaccination status. The middle panel shows the ACM rate by vaccination status for each month between January 2020 and December 2022, the average ACM rate disregarding the vaccination status (black line) and the expected ACM rate (green line, see the Methods for details on the calculation). The bottom panel shows the ACM rates relative to the ACM rate of the unvaccinated. Vaccination status is color-coded as follows: Unvaccinated – red; individuals after a single dose of any COVID-19 vaccine – dark blue; individuals after two doses of any COVID-19 vaccine – blue; individuals after three or more doses – light blue. Note that mass vaccination for this group started on March 1, 2021; before that date, only the frailest individuals highlighted for preferential vaccination received the vaccine

A set of figures was generated for each age cohort (i.e., individuals born before 1980). In the main body of this manuscript, we present figures for men and women born between 1940 and 1949, as this cohort includes the most deaths (*N* = 118,668) in the dataset. The charts for both sexes are presented separately (Figs. 1 and 2) due to the differences in mortality patterns between men and women across age cohorts. Figures describing the ACM evolution for both sexes and all remaining age brackets are presented as Supplementary Figs. 1–10. The computed numbers of deaths and person-years used to produce these graphs are available in the Supplementary material as well.

Figures 1 and 2 provide a wealth of information; however, their interpretation is not completely straightforward. In the spring and summer of 2021, the ACM was several times higher among unvaccinated compared to those who were vaccinated. This was followed by a further decline in mortality of those vaccinated with the booster dose compared to those who remained vaccinated with the primary course only. Conversely, ACM was notably high among individuals vaccinated with the first dose in the beginning of 2021. The ACM among individuals who received only the first vaccine dose was persistently high from mid-2021 until the end of the study. Furthermore, a peculiar pattern emerged, suggesting that COVID-19 vaccines conferred a protective effect against death even during the summer of 2021, when practically no COVID-19-related deaths were reported in the Czech Republic.

The observed patterns for men and women were highly consistent across cohorts, although ACM was generally lower among females throughout the entire study period.

## Discussion

This study aimed to investigate patterns of all-cause mortality (ACM) using population-wide data from the Czech Republic, and to discuss the possible causes and implications of these patterns. The interpretation of the findings is difficult: as the Mirror of Erised in Harry Potter books, the findings offer everyone a reflection of the pandemic they desire to see.

### High mortality of individuals after a single dose

In principle, there are two periods when the mortality of individuals with a single dose of COVID-19 vaccines was higher than that of the unvaccinated population: before the beginning of mass vaccination of this cohort (March 2021) and from summer 2021 onwards. Vaccine sceptics may interpret this as evidence of vaccine-induced mortality; however, other factors are in play here: in the first period, we must consider the indication bias, i.e., the preferential vaccination of the frailest individuals in care homes or those who were chronically ill. It is, therefore, not surprising that the mortality rate in this very small group (see the top panel in Figs. 1 and 2) was very high. Once mass vaccination was open to everyone in that cohort, the ACM plummeted, which could be explained by the influx of relatively healthy individuals and, therefore, the “dilution” of the previously high concentration of frail individuals. However, over a few months, most of those whose health was good went on to receive the second dose of the vaccine, leaving only a very small fraction of the population with a single dose – and in this population, the frail individuals (unable to receive the second dose) concentrated again. This is a likely explanation of the observed pattern.

### Low mortality of the vaccinated in spring and summer 2021

At first sight, it may appear that the very low mortality of those vaccinated with the primary course (two doses) is a proof of high vaccine effectiveness. However, multiple caveats need to be considered.

First of all, even a 100% vaccine effectiveness against COVID-related death could not cause a massive drop in all-cause mortality which was observed in April and May 2021 when ACM for those vaccinated with two doses was less than 1/5 (16⋅9% and 19⋅7% respectively, i.e. more than 80% drop in ACM) compared to unvaccinated, suggesting a major selection/indication bias. Moreover, as mass vaccination for this cohort only started in March, the vast majority of this cohort was double-vaccinated only in April when the COVID-19 wave was already dwindling. Taking this a step further, in the summer months (June to September 2021), there were practically no reported COVID-19-related deaths in the Czech Republic (less than 100 COVID-19-related deaths out of approx. 35,000 total deaths in this period for the whole country) – and yet, a profound difference in ACM was observed between unvaccinated and those vaccinated with two doses. This apparent protection against non-COVID deaths conveyed by the vaccines suggests a massive selection/indication bias (Healthy Vaccinee Effect, HVE).

One may speculate that some deaths in these months occurred due to previous COVID-19 infection, and that the difference in ACM in that period may be caused by the fact that the vaccinated enjoyed some protection against post-COVID-19 sequelae. However, this hypothesis does not stand under scrutiny as the total ACM in these months perfectly fits the one predicted based on the mortality from 2019 (i.e., the black and green lines in the medium panels of Figs. 1 and 2 overlay almost perfectly in the summer of 2021). Had the post-COVID sequelae been the cause, the ACM in summer 2021 would have been much higher than that predicted based on 2019. Moreover, note that vaccination started in this cohort only at the end of the COVID-19 wave so it could not have prevented COVID-19 on such a massive scale that would explain the magnitude of differences in ACM between the unvaccinated and those vaccinated with the primary course. Hence, the apparent effectiveness of the vaccines in summer 2021 must be attributed to profound structural differences between the group who chose to get vaccinated, and the group who chose to abstain.

As presented above, the risk of death from non-COVID causes was up to five times lower among vaccinated individuals during periods with negligible COVID-19 mortality. This implies a risk ratio (entirely attributable to the HVE) close to 0.2, corresponding to an apparent vaccine effectiveness of approximately 80% against non-COVID mortality. Such a magnitude is comparable to some observational estimates of vaccine effectiveness, but is still considerably lower than others, particularly those reported against COVID-19 mortality during the Delta period, which often exceeded 95–99%. This highlights the importance of interpreting high vaccine effectiveness estimates, particularly those exceeding 90%, with caution, as they may reflect both true vaccine benefit and selection bias. These concerns are supported by prior studies (e.g., Høeg et al. [13]), which reported similar reductions in both COVID-related and unrelated mortality. While methods to adjust for HVE are available, they rely on assumptions that warrant careful examination.

### The booster campaign

For interpretation, it is necessary to point out that the booster campaign started practically simultaneously with the delta wave (turn of October/November 2021). There was, unfortunately, no period of booster vaccination preceding the delta wave, which could be used to estimate the magnitude of the HVE during the booster campaign. We can, however, observe a clear rise in the mortality of double-vaccinated individuals at the time the booster campaign started. This may again be caused by the HVE, similar to what we observed in the primary course of vaccination: the healthier part of the double-vaccinated population and those who took generally better care for their health went on to receive the booster dose (e.g. ACM of boosted men was only 29⋅3% and 20⋅3% of double vaccinated men without a booster in November and December 2021, respectively). This is further corroborated by the fact that by early 2022, the mortality of those with two doses and the unvaccinated ones evened out and remained on par until the end of the study period, while the mortality of those with the booster dose was constantly much lower, even after the COVID-19 wave ended in April 2022.

The results for the rest of the age cohorts (presented as Supplementary material) were consistent with those described here, differing predominantly in the date the vaccination campaign for the respective cohort started and, therefore, in the moment when indication bias was overwhelmed by the Healthy Vaccinee bias.

These findings have profound implications for studies assessing vaccine effectiveness and safety that rely on observation data: The structural differences between the cohort choosing to get vaccinated and the cohort choosing to abstain were substantial, leading to a surprisingly large and persistent disparity in ACM rates between the two groups. These differences were present, of course, both during and outside epidemic waves, complicating efforts to produce reliable estimates of COVID-19 vaccine effectiveness from observational studies. Notably, studies that fail to account for these structural differences may present an inflated estimate of vaccine effectiveness.

### Addressing bias and contextualizing the findings

Our study highlights the persistence of well-known biases – particularly selection/indication bias – in observational analyses of vaccine effectiveness. While these biases have been acknowledged in the literature, our contribution lies in the quantification of their magnitude and persistence using a unique, openly available, individual-level dataset covering the entire Czech population. This level of transparency and coverage is rarely available and enables reproducibility of the findings.

Since the dataset does not contain the cause of death, we were unable to provide any (uncorrected or corrected) estimates of vaccine effectiveness against COVID-related death. Other studies have attempted to mitigate similar biases using various strategies, such as incorporating confounders including demographic and comorbidity data [14, 15], employing self-controlled case series designs [16], focusing on laboratory-confirmed COVID-19 deaths [15, 17], or implementing inverse probability weighting [14]. These methods, however, require detailed individual health information or other data not available in our setting. We hope that the level of transparency in the Czech Republic data will encourage other public health authorities to publish similar datasets.

Importantly, the observed patterns in our study are consistent with findings from other countries. For example, studies by Høeg et al. [13], Hulme et al. [18], and Liu et al. [15] reported similar distortions in vaccine effectiveness estimates when using all-cause mortality as an outcome. Our findings also align with patterns reported in official statistics from the UK Office for National Statistics [19]. This broader context underscores the challenges of using administrative data to evaluate vaccine effectiveness and the importance of careful methodological choices in such analyses.

### Strengths and limitations

In this study, we have used a nationwide dataset containing over 5 million records. A key strength of this study is that the dataset is publicly available, allowing for the reproduction and verification of our results. The fact that we use ACM only can be considered both a strength and a limitation: the strength lies in the reliability of data that is not burdened with any testing biases, false positives, or false negatives; on the other hand, the inability to distinguish between COVID-19 and non-COVID-19 deaths within the same dataset limits our capacity to estimate the magnitude of the Healthy Vaccinee Effect. As such, our aim is not to provide adjusted estimates of vaccine effectiveness, but rather to demonstrate the presence and persistence of biases in unadjusted administrative data. Another limitation of this study is the absence of key individual-level variables, such as comorbidities, which would have enhanced the interpretability of our findings. Unfortunately, such data was not available to us. Finally, although the nationwide dataset used in this study is both comprehensive and openly accessible, this level of data availability may not be achievable in other countries due to legal, ethical, or institutional constraints. As a result, the generalizability of our approach may be limited by differences in data governance and health system structures across countries.

## Conclusions

In this paper, we have described the associations between the all-cause mortality rates and COVID-19 vaccination status in the Czech population. The data suggest the existence of multiple biases in the observational data – besides the indication bias observed particularly at the beginning of the campaign, a substantial Healthy Vaccinee bias was observed as well. Failure to account for this bias represents a major problem for observational studies on COVID-19 vaccine effectiveness and safety.

## Supplementary Information

Supplementary Material 1.

## Data Availability

The datasets generated and/or analyzed during the current study are available in the repository https://github.com/PalackyUniversity/uzis-data-analysis. For more information, see the Supplementary material, Sect. 1.

## References

[1] Cromer D, Steain M, Reynaldi A, Schlub TE, Khan SR, Sasson SC, Kent SJ, Khoury DS, Davenport MP. Predicting vaccine effectiveness against severe COVID-19 over time and against variants: a meta-analysis. Nat Commun. 2023;14(1). 10.1038/s41467-023-37176-7. 10.1038/s41467-023-37176-7 PMC10036966 36964146

[2] Feikin DR, Higdon MM, Abu-Raddad LJ, Andrews N, Araos R, Goldberg Y, Groome MJ, Huppert A, O’Brien KL, Smith PG, et al. Duration of effectiveness of vaccines against SARS-CoV-2 infection and COVID-19 disease: results of a systematic review and meta-regression. Lancet (London England). 2022;399(10328):924–44. 10.1016/S0140-6736(22)00152-0. 35202601 10.1016/S0140-6736(22)00152-0 PMC8863502

[3] Soheili M, Khateri S, Moradpour F, Mohammadzedeh P, Zareie M, Mortazavi SMM, Manifar S, Kohan HG, Moradi Y. The efficacy and effectiveness of COVID-19 vaccines around the world: a mini-review and meta-analysis. Ann Clin Microbiol Antimicrob. 2023;22(1):42. 10.1186/s12941-023-00594-y. 37208749 10.1186/s12941-023-00594-y PMC10198032

[4] Tanveer S, Rowhani-Farid A, Hong K, Jefferson T, Doshi P. Transparency of COVID-19 vaccine trials: decisions without data. Bmj Evidence-Based Med. 2022;27(4):199–205. 10.1136/bmjebm-2021-111735. 10.1136/bmjebm-2021-111735 34373256

[5] Ioannidis JPA. Factors influencing estimated effectiveness of COVID-19 vaccines in non-randomised studies. Bmj Evidence-Based Med. 2022;27(6):324–9. 10.1136/bmjebm-2021-111901. 10.1136/bmjebm-2021-111901 PMC9691814 35338091

[6] Institute of the Health Information and Statistics of the Czech Republic. [https://www.uzis.cz/index-en.php]. Accessed 27 Nov 2024.

[7] Fürst T, Bazalová A, Frycák T, Janosek J. Does the healthy vaccinee bias rule them all? Association of COVID-19 vaccination status and all-cause mortality from an analysis of data from 2.2 million individual health records. Int J Infect Dis. 2024;142. 10.1016/j.ijid.2024.02.019. 10.1016/j.ijid.2024.02.019 38401782

[8] Furst T, Straka R, Janosek J. Healthy vaccinee effect: a bias not to be forgotten in observational studies on COVID-19 vaccine effectiveness. Pol Archives Intern Med. 2024;134(2):16634–16634. 10.20452/pamw.16634. 10.20452/pamw.16634 38415516

[9] Straka R, Furst T, Janosek J. Healthy vaccinee effect in practice. Pol Archives Intern Med. 2024;134(5):16750–16750. 10.20452/pamw.16750. 10.20452/pamw.16750 38804815

[10] Remschmidt C, Wichmann O, Harder T. Frequency and impact of confounding by indication and healthy vaccinee bias in observational studies assessing influenza vaccine effectiveness: a systematic review. BMC Infect Dis. 2015;15. 10.1186/s12879-015-1154-y. 10.1186/s12879-015-1154-y PMC4609091 26474974

[11] Czech Statistical Office. Complete life tables– 2019 [https://vdb.czso.cz/vdbvo2/faces/en/index.jsf?page=vystup-objekt-parametry&z=T&f=TABULKA&katalog=32592&pvo=DEMD003-CR&sp=A&skupId=2462&c=v3%7E8__RP2019&str=v136]. Accessed 27 Nov 2024.

[12] Czech Statistical Office. Czech Demographic Handbook– 2022 [https://csu.gov.cz/produkty/czech-demographic-handbook-2022]. Accessed 27 Nov 2024.

[13] Høeg TB, Duriseti R, Prasad V. Potential healthy vaccinee Bias in a study of BNT162b2 vaccine against Covid-19. N Engl J Med. 2023;389(3):284–5. 10.1056/NEJMc2306683. 37470285 10.1056/NEJMc2306683

[14] Xu S, Huang R, Sy LS, Hong V, Glenn SC, Ryan DS, Morrissette K, Vazquez-Benitez G, Glanz JM, Klein NP, et al. A safety study evaluating non-COVID-19 mortality risk following COVID-19 vaccination. Vaccine. 2023;41(3):844–54. 10.1016/j.vaccine.2022.12.036. 36564276 10.1016/j.vaccine.2022.12.036 PMC9763207

[15] Liu B, Stepien S, Dobbins T, Gidding H, Henry D, Korda R, Mills L, Pearson SA, Pratt N, Vajdic CM, et al. Effectiveness of COVID-19 vaccination against COVID-19 specific and all-cause mortality in older australians: a population based study. Lancet Reg Health Western Pac. 2023;40:100928. 10.1016/j.lanwpc.2023.100928. 10.1016/j.lanwpc.2023.100928 PMC10579525 37854458

[16] Xu S, Sy LS, Hong V, Farrington P, Glenn SC, Ryan DS, Shirley AM, Lewin BJ, Tseng HF, Vazquez-Benitez G, et al. Mortality risk after COVID-19 vaccination: A self-controlled case series study. Vaccine. 2024;42(7):1731–7. 10.1016/j.vaccine.2024.02.032. 38388239 10.1016/j.vaccine.2024.02.032 PMC11238073

[17] Andersson NW, Thiesson EM, Pihlström N, Perälä J, Faksová K, Gram MA, Poukka E, Leino T, Ljung R, Hviid A. Comparative effectiveness of monovalent XBB.1.5 containing covid-19 mRNA vaccines in denmark, finland, and sweden: target trial emulation based on registry data. BMJ Med. 2024;3(1):e001074. 10.1136/bmjmed-2024-001074. 39902239 10.1136/bmjmed-2024-001074 PMC11789462

[18] Hulme WJ, Williamson E, Horne EMF, Green A, McDonald HI, Walker AJ, Curtis HJ, Morton CE, MacKenna B, Croker R, et al. Challenges in estimating the effectiveness of COVID-19 vaccination using observational data. Ann Intern Med. 2023;176(5):685–93. 10.7326/M21-4269. 37126810 10.7326/M21-4269 PMC10152408

[19] Office for National Statistics. Deaths by vaccination status, England. [https://www.ons.gov.uk/peoplepopulationandcommunity/birthsdeathsandmarriages/deaths/datasets/deathsbyvaccinationstatusengland]. Accessed 5 May 2025.

