# Supplementary material for: The Mirror of Erised: a retrospective population-wide study of Czech all-cause mortality data by COVID-19 vaccination status
Source: BMC Public Health. 2025 Jul 10;25:2427. doi: 10.1186/s12889-025-23619-x (PMC12243391; doi:10.1186/s12889-025-23619-x)

The Mirror of Erised: Czech Mortality Data by COVID-19 Vaccination Status

Supplementary Material

Contents

1. Underlying data ... 1
2. Supplementary Table S1 ... 3
   Table S1 ... 4
3. Supplementary Figures SF1– SF10. ... 5
   Figure SF1 ... 6
   Figure SF2 ... 7
   Figure SF3 ... 8
   Figure SF4 ... 9
   Figure SF5 ... 10
   Figure SF6 ... 11
   Figure SF7 ... 12
   Figure SF8 ... 13
   Figure SF9 ... 14
   Figure SF10 ... 15

1. Underlying data

The repository https://github.com/PalackyUniversity/uzis-data-analysis contains the folder "Source-data" which contains CSV files with the data needed to reconstruct the figures described in the Manuscript and in the *Supplementary Figures SF1–SF10*. Each CSV file is provided in two mutations: (1) an English version (e.g. "group_born1925-1929-M-data.csv") with comma separated values, decimal points, and English headers, and (2) a Central

European version (e.g. “CZ-group_born1925-1929-M-data.csv”) with identical numbers but semicolon separated values, decimal commas, and English headers. The sex and age cohort is described in the filename.

- Each “*-data.csv” file contains the following fields:

  Mon: Identification of the month

  Status: Vaccination status

  person_days: Number of person-days spent by individuals of the respective sex, age, and vaccination status in that month

  Deaths: Number of individuals of the respective sex, age, and vaccination status that died during the month from any cause

  MortalityRate: All-cause mortality rate as the ratio of *Deaths* (field 4 above) and *person_days* (field 3 above) converted to the standard unit – deaths per thousand person-years.

  MR_unvac: All-cause mortality rate of the unvaccinated (given sex, age cohort, and month)

  person_days_AllGroups: Number of person-days spent by individuals of the respective sex, age in that month (regardless of vaccination status)

  Deaths_AllGroups: Number of individuals of the respective sex, age (regardless of vaccination status) that died during the month from any cause

  Average_mortality_rate: All-cause mortality rate in the given month for given sex and age group: average over all groups defined by vaccination status. It is computed as the ratio of *Deaths_AllGroups* (field 8 above) and *person_days_AllGroups* (field 7 above) converted to the standard unit – deaths per thousand person-years.

  MR_ComparedToUnvacc: All-cause mortality rate (given sex, age cohort, and vaccination status) divided by the all-cause mortality rate of the unvaccinated (given sex and age cohort)

  GroupSize_Share: Proportion of person-days corresponding to the particular vaccination status (given sex, age cohort, and month)

- Each “*-pred.csv” file contains the following fields:

  Year

  mon: month

  Mon: Year and month together

  Quantity: ‘Expected’, ‘Upper’, or ‘Lower’. Extent of the green boxes in the figures, see Methods for details

  P: Proportion of deaths in that year that are expected to occur in the given month (sum of the values of *p* over 12 months of a year equals to 1).

  year.total: Expected number of deaths in the respective sex, age-cohort and year

  At_risk_NY: Total number of individuals of the respective sex and age-cohort that were alive on Jan 1 of the year

  Count: Expected number of deaths in the respective sex, age cohort, and month

  died.before: Expected cumulative number of individuals of the respective sex and age cohort that died since the beginning of the year

  At_risk : Expected total number of individuals of the respective sex and age-cohort alive at the beginning of the month

  Days: Length of the month

  MR: Expected all-cause mortality (given sex, age cohort and month) converted to the standard unit – deaths per thousand person-years. See Methods for details of the computation.

2. Supplementary Table S1

Table S1. A detailed breakdown of COVID-19 vaccine types and doses in the Czech population*;* *Czec*h Republic, 2020–2022.

	**dose1**	**dose2**	**dose3**	**dose4**	**dose5**	**dose6**	**dose7**
**UNVACCINATED**	**4,046,234**	**4,544,448**	**6,650,640**	**10,117,311**	**10,738,090**	**11,026,575**	**11,028,323**
CO01 COMIRNATY	5,586,979	5,519,975	3,747,813	282,233	13,876	66	1
CO02 SPIKEVAX	526,453	517,783	568,220	22,353	142	6	0
CO03 VAXZEVRIA	447,450	439,705	433	13	0	0	0
CO04 COVID-19 VACCINE JANSSEN	412,314	271	2,463	39	1	0	0
CO05 SPUTNIK V	10	10	2	1	0	0	0
CO06	0	0	0	0	0	0	0
CO07 NUVAXOVID	5,425	5,098	291	423	63	5	0
CO08 COMIRNATY ORIGINAL/OMICRON BA.1	293	230	11,141	107,479	2,149	13	0
CO09 COMIRNATY ORIGINAL/OMICRON BA.4/BA.5	665	300	39,250	390,358	6,215	43	0
CO10 COVOVAX	29	29	0	0	0	0	0
CO11 SINOPHARM	38	39	12	0	1	0	0
CO12 SINOVAC	180	178	9	1	0	0	0
CO13 COVAXIN	5	4	1	0	0	0	0
CO14 COVISHIELD	115	94	3	0	0	0	0
CO15 SPIKEVAX BIVALENT ORIGINAL/OMICRON BA.1	15	16	862	7,499	85	0	0
CO16 COMIRNATY 6M-4	111	92	64	0	0	0	0
CO17 VALNEVA	2	2	0	0	0	0	0
CO18	0	0	0	0	0	0	0
CO19 SPIKEVAX BIVALENT ORIGINAL/OMICRON BA.4-5	4	2	12	64	107	1	0
CO20 COMIRNATY OMICRON XBB.1.5.	1,924	67	6,918	99,200	264,645	1,647	47
CO21 COMIRNATY OMICRON XBB.1.5. 5-11	97	2	197	1,205	2,844	16	1
CO22 NUVAXOVID XBB 1.5	3	2	25	183	145	0	0
CO23 COMIRNATY OMICRON XBB.1.5 6M-4	26	25	16	10	9	0	0

3. Supplementary Figures SF1– SF10.

Figures SF1–SF10: The all-cause mortality (ACM) figures for both sexes and all relevant age cohorts; Czech Republic, 2020–2022.

The repository https://github.com/PalackyUniversity/uzis-data-analysis contains the folder "ACM-figures" with all the relevant figures.

Each figure shows the evolution of the all-cause mortality rate in the respective cohort. The top panel shows the relative composition of the cohort according to the vaccination status. The middle panel shows the ACM rate by vaccination status for each month between January 2020 and December 2022. The average ACM rate (disregarding the vaccination status) is shown in black and the expected ACM rate (see the Methods for details on the calculation) is shown by the green boxes. The bottom panel shows the ACM rates relative to the ACM rate of the unvaccinated. Vaccination status is color-coded as follows: Unvaccinated – red; individuals after a single dose of any COVID-19 vaccine – dark blue; individuals after two doses of any COVID-19 vaccine – blue; individuals after three or more doses – light blue.

Figure SF1. Evolution of the all-cause mortality (ACM) rate in the cohort of women born between 1925 and 1929; Czech Republic, 2020–2022.

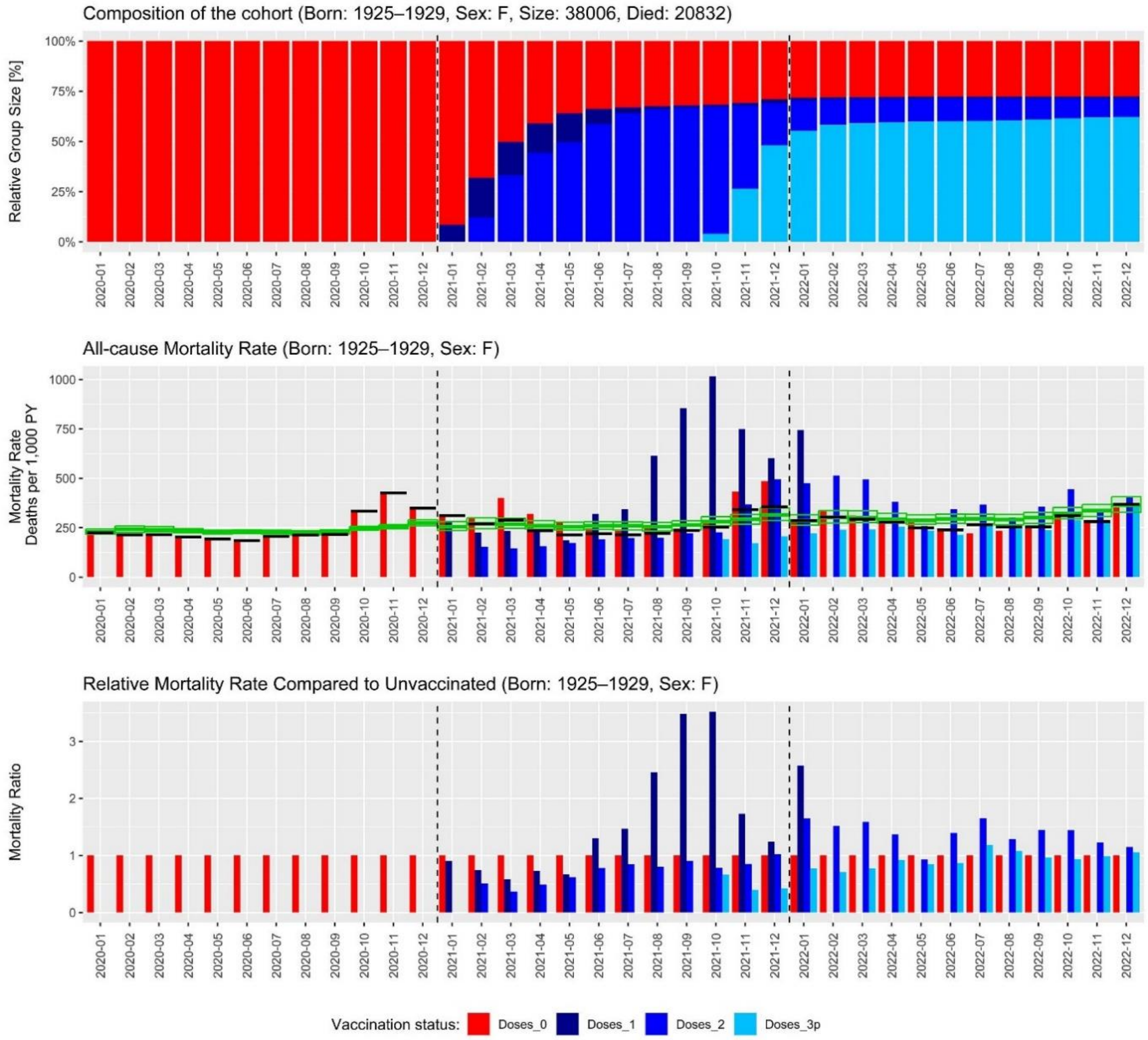

Figure SF2. Evolution of the all-cause mortality (ACM) rate in the cohort of men born between 1925 and 1929; Czech Republic, 2020–2022.

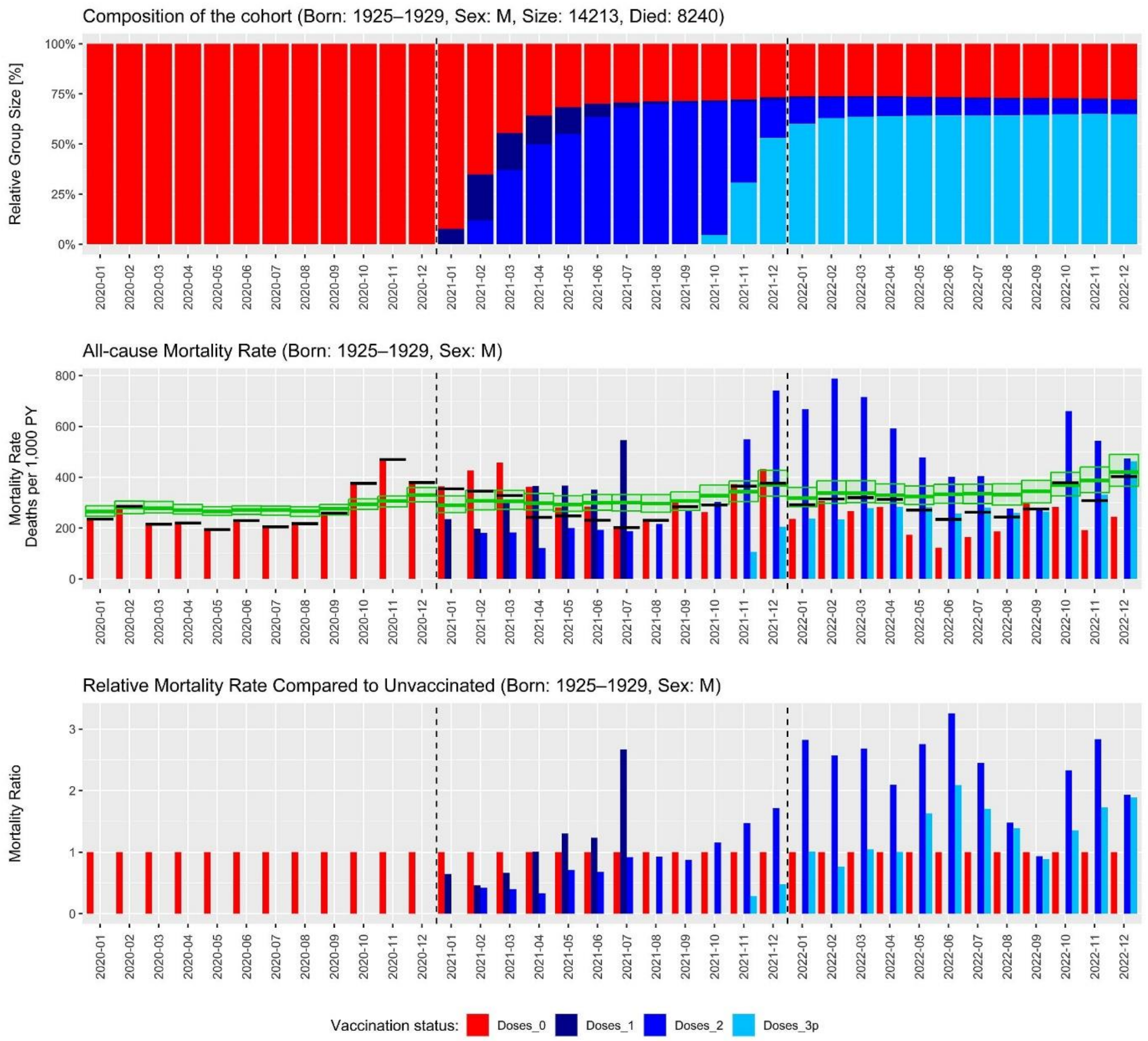

Figure SF3. Evolution of the all-cause mortality (ACM) rate in the cohort of women born between 1930 and 1939; Czech Republic, 2020–2022.

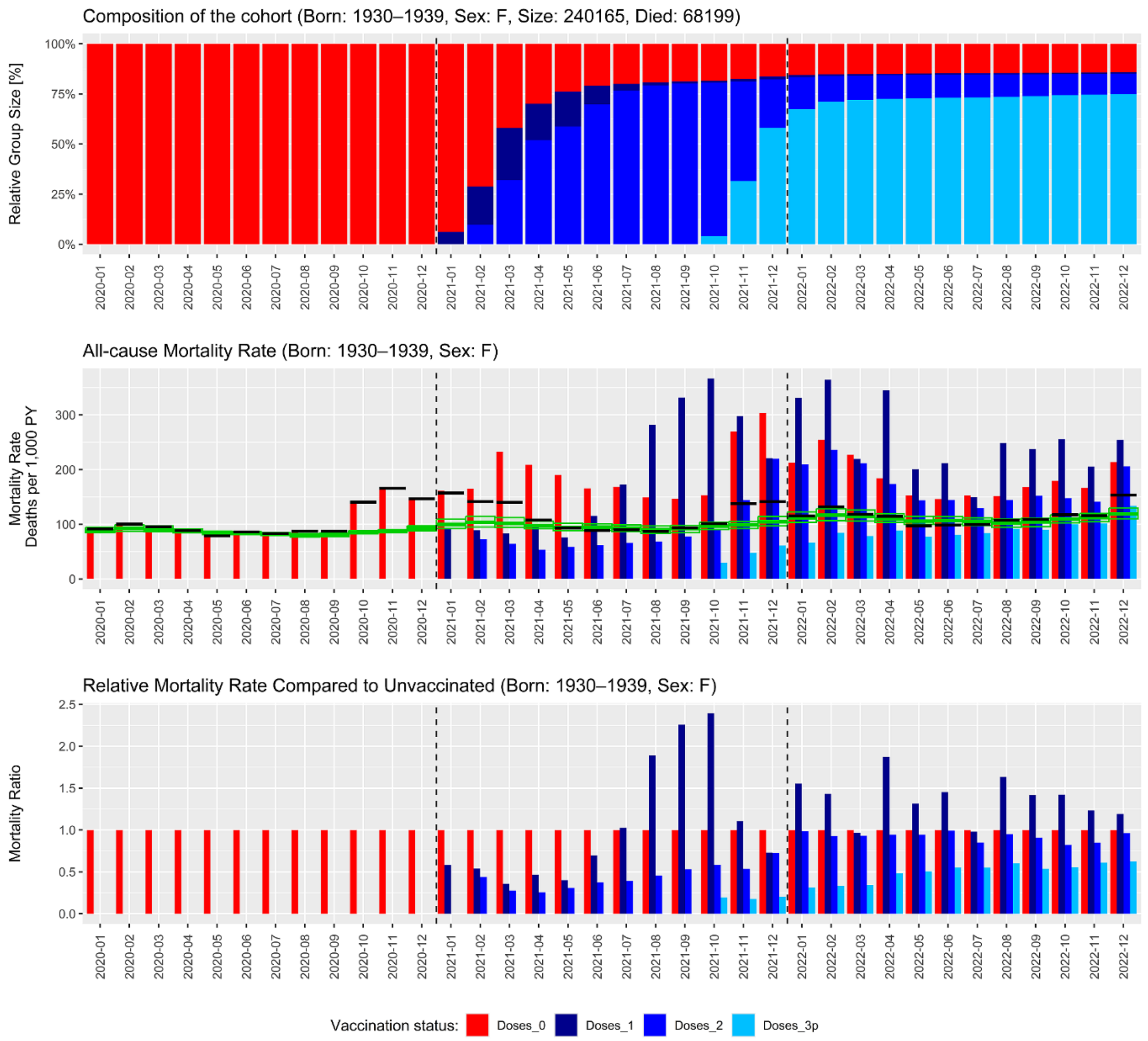

Figure SF4. Evolution of the all-cause mortality (ACM) rate in the cohort of men born between 1930 and 1939; Czech Republic, 2020–2022.

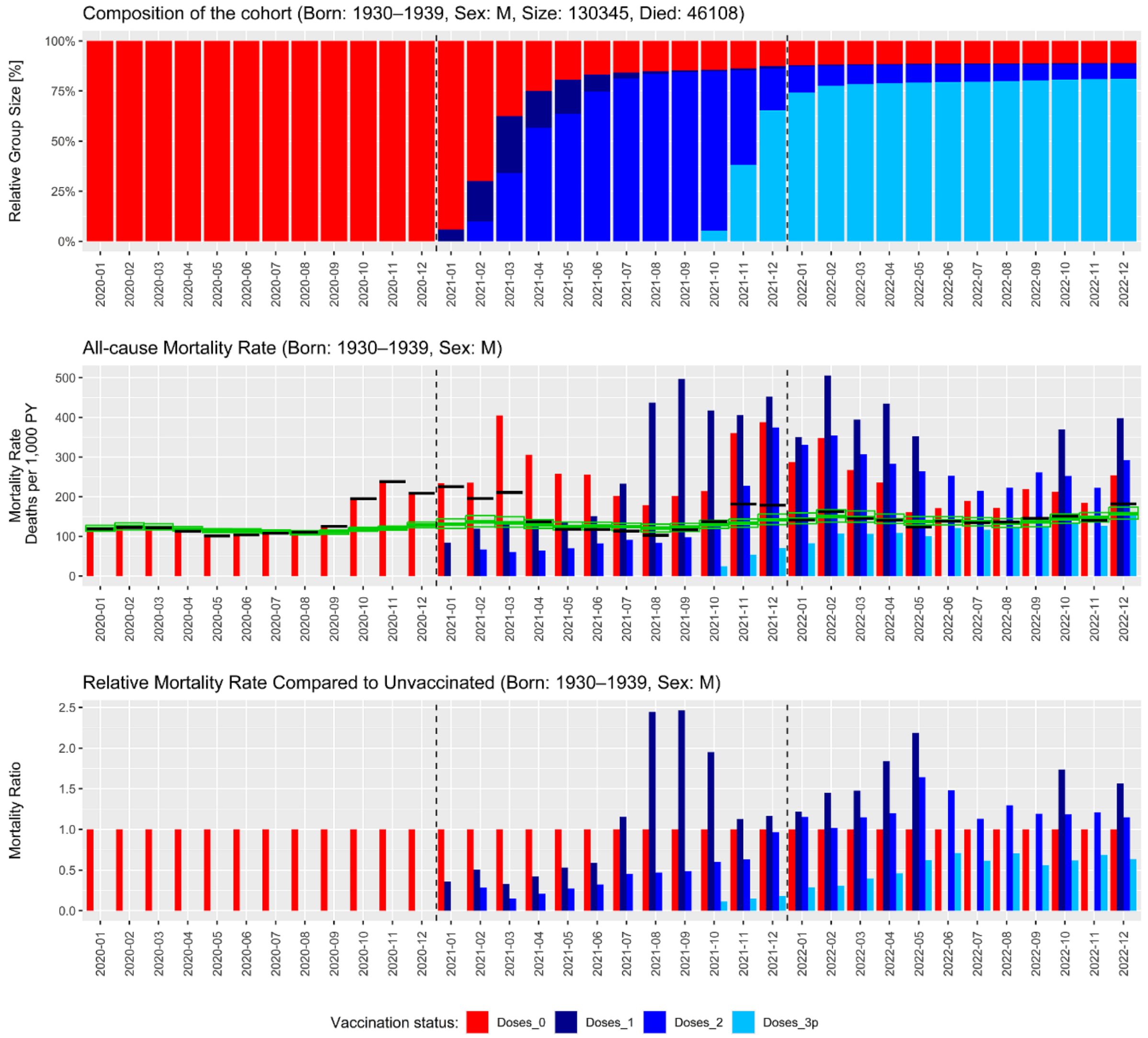

Figure SF5. Evolution of the all-cause mortality (ACM) rate in the cohort of women born between 1950 and 1959; Czech Republic, 2020–2022.

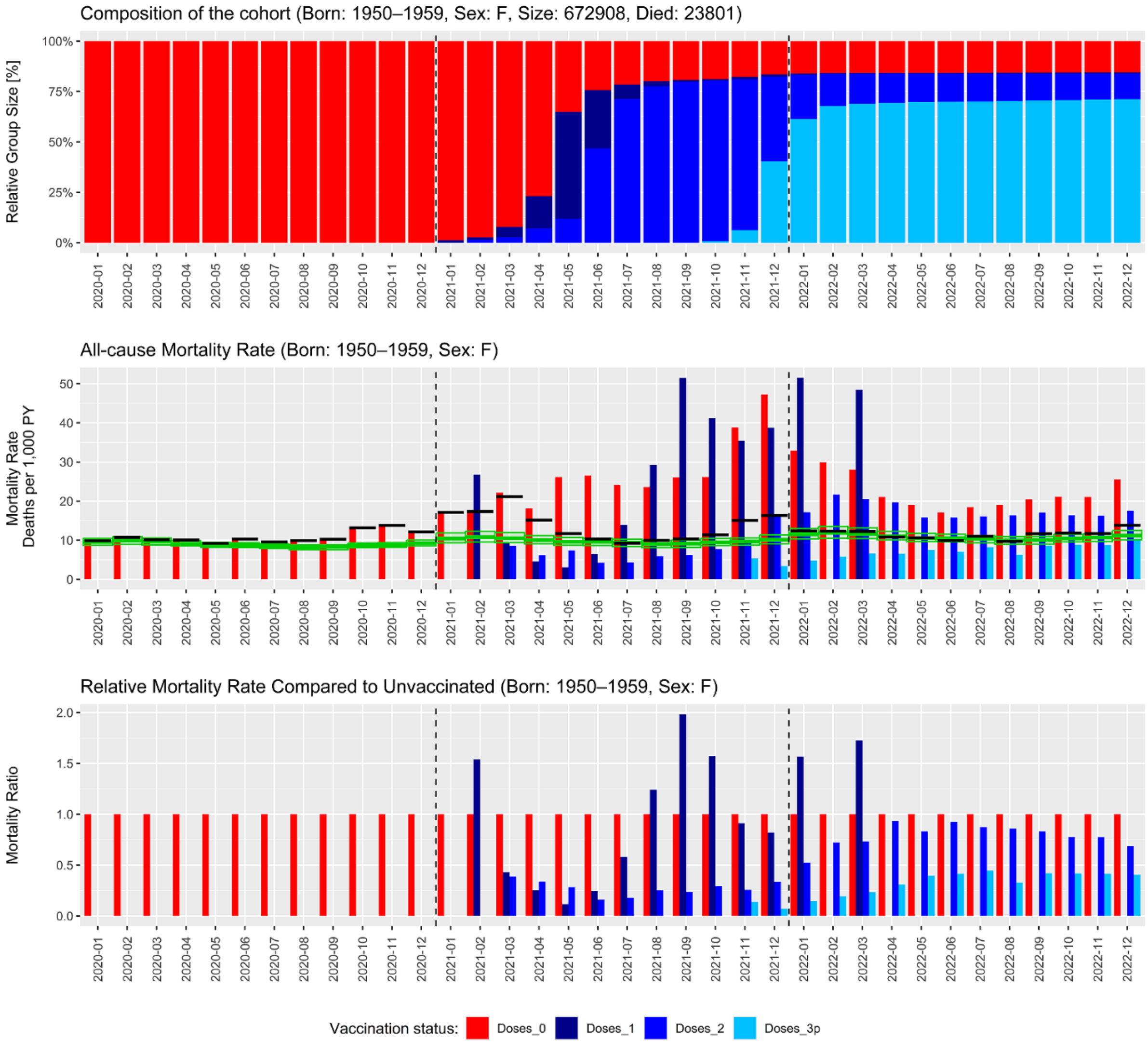

Figure SF6. Evolution of the all-cause mortality (ACM) rate in the cohort of men born between 1950 and 1959; Czech Republic, 2020–2022.

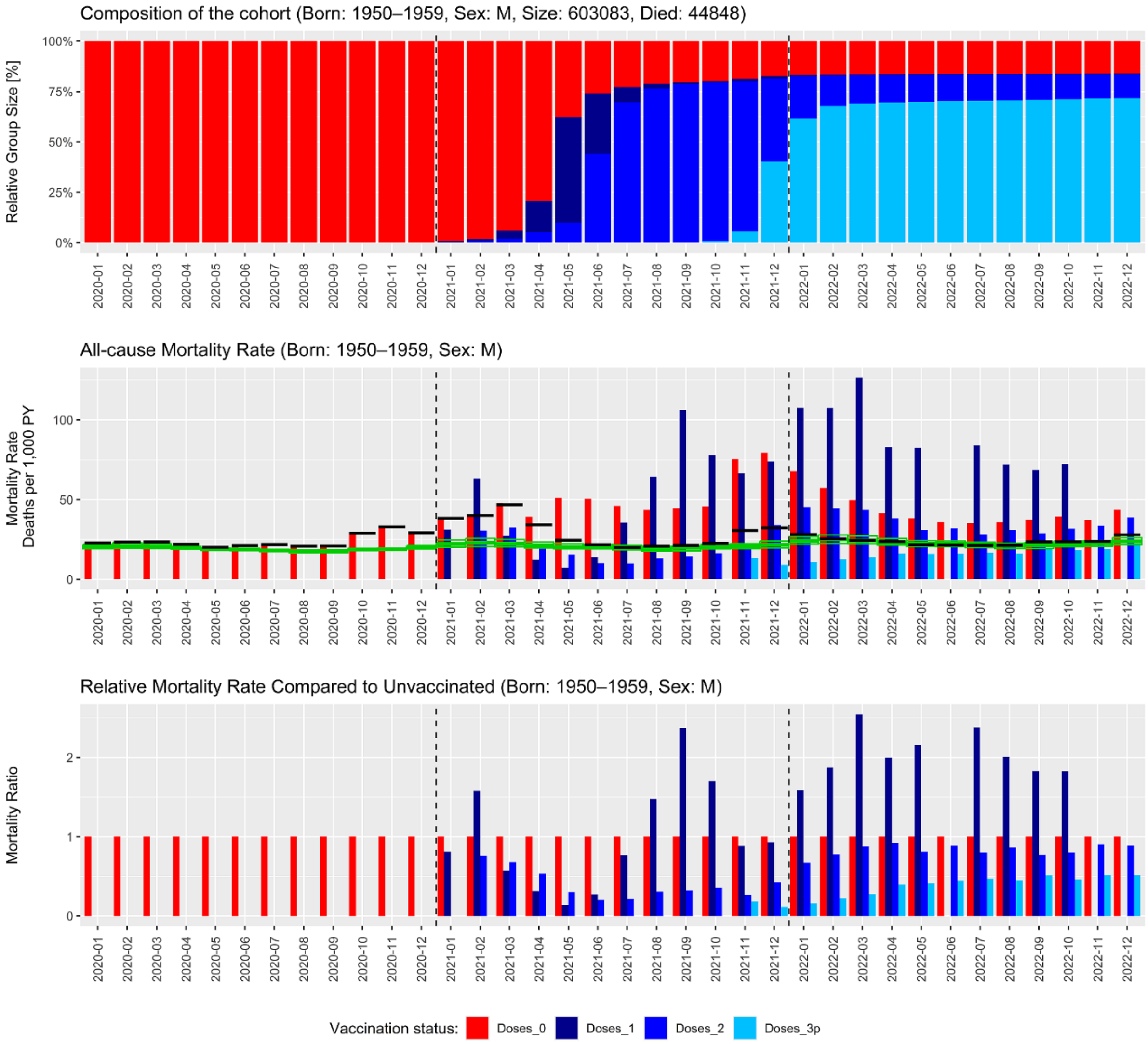

Figure SF7. Evolution of the all-cause mortality (ACM) rate in the cohort of women born between 1960 and 1969; Czech Republic, 2020–2022.

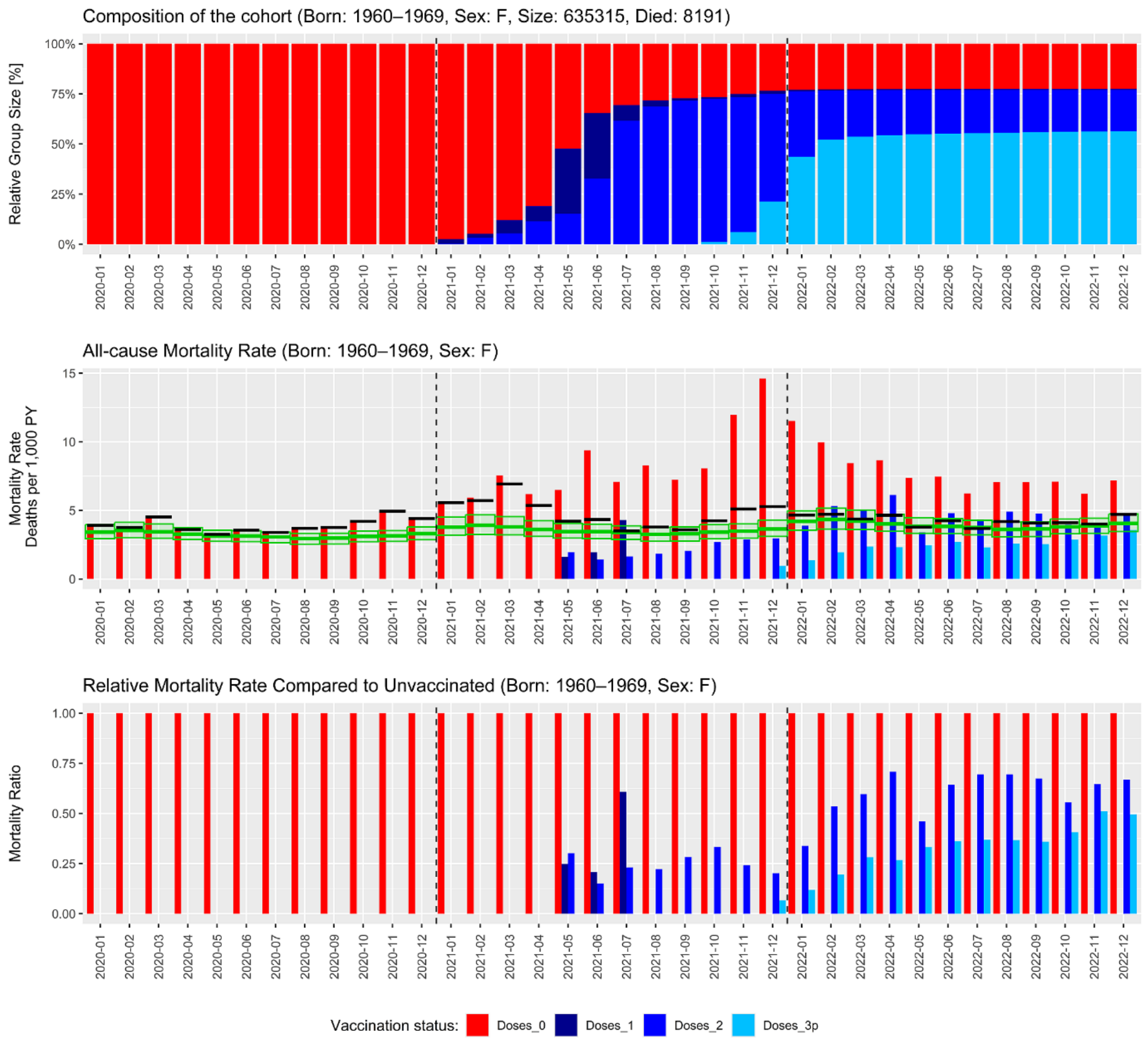

Figure SF8. Evolution of the all-cause mortality (ACM) rate in the cohort of men born between 1960 and 1969; Czech Republic, 2020–2022.

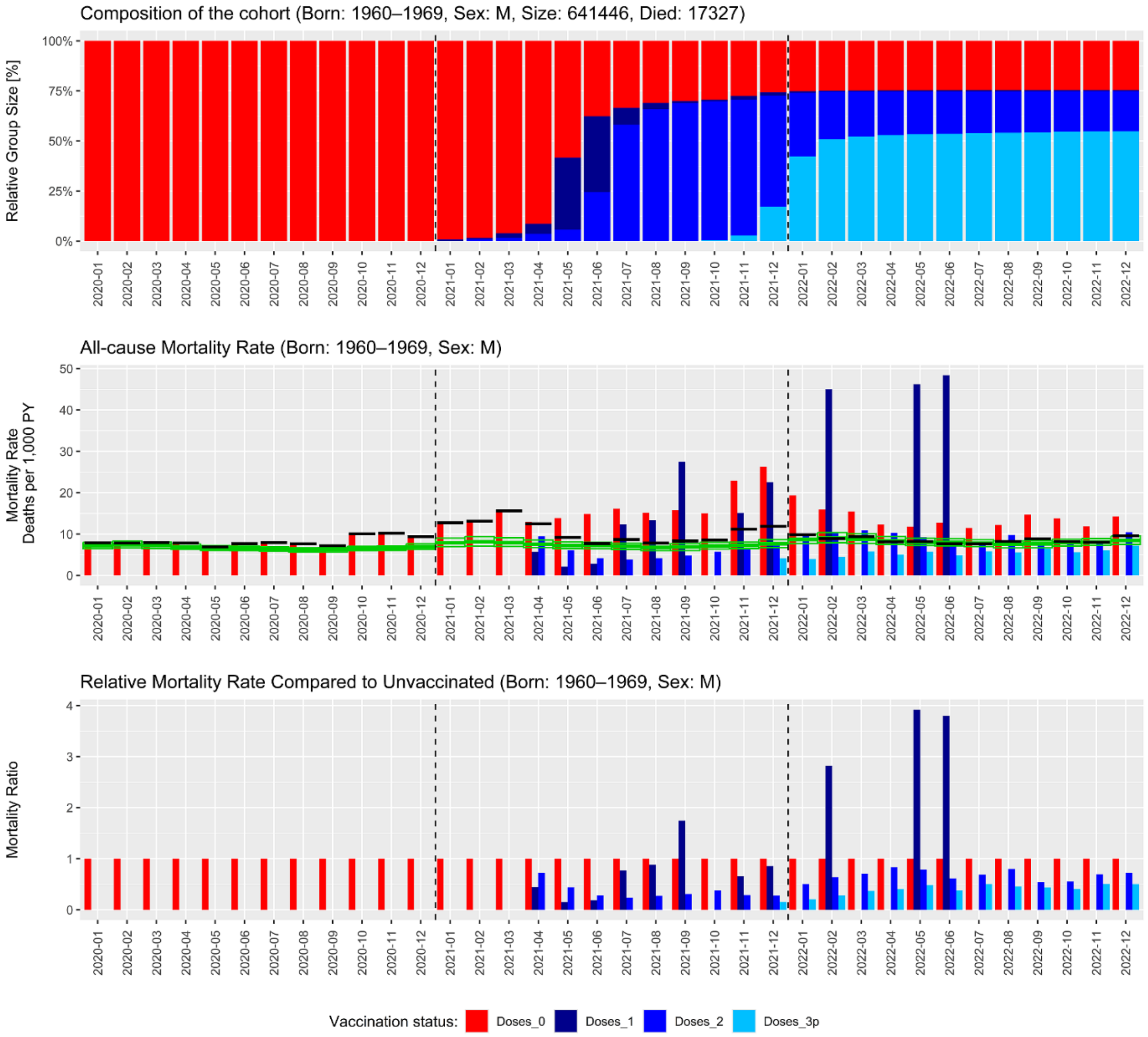

Figure SF9. Evolution of the all-cause mortality (ACM) rate in the cohort of women born between 1970 and 1979; Czech Republic, 2020–2022.

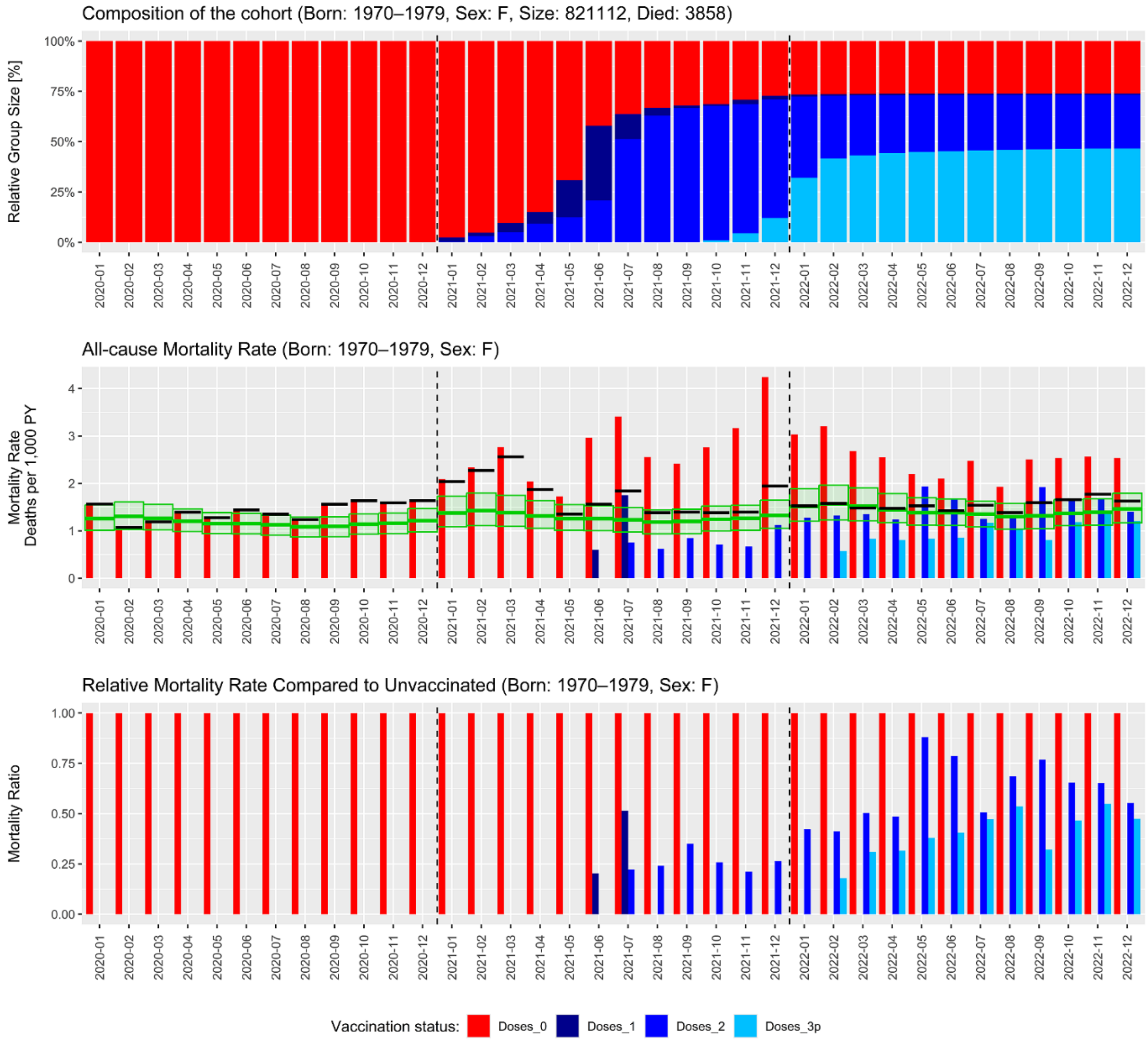

Figure SF10. Evolution of the all-cause mortality (ACM) rate in the cohort of men born between 1970 and 1979; Czech Republic, 2020–2022.

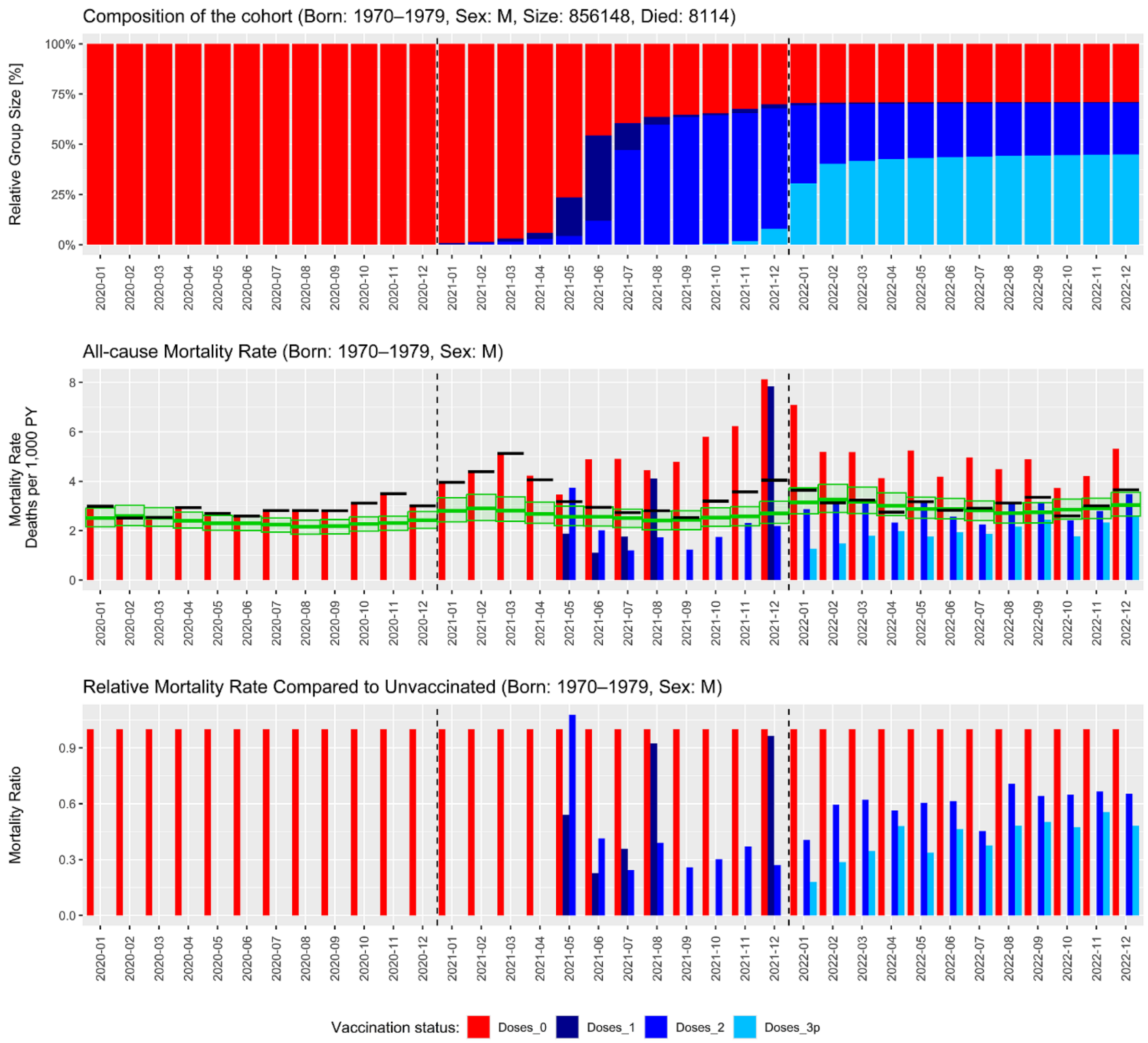

Supplement: Supplementary file 1 — Supplementary Material 1. [file 12889_2025_23619_MOESM1_ESM.pdf]
